# Responsiveness of electromyographically assessed skeletal muscle inactivity: methodological exploration and implications for health benefits

**DOI:** 10.1038/s41598-022-25128-y

**Published:** 2022-12-02

**Authors:** A. J. Pesola, Y. Gao, T. Finni

**Affiliations:** 1 Active Life Lab, South-Eastern Finland University of Applied Sciences, Raviradantie 22b, 50100 Mikkeli, Finland; 2 Department of Sports Science, College of Education, Zhejiang University, Hangzhou, China; 3 Faculty of Sport and Health Sciences, Neuromuscular Research Center, University of Jyväskylä, Jyväskylä, Finland

**Keywords:** Skeletal muscle, Risk factors, Randomized controlled trials

## Abstract

Prolonged sedentary behaviour is detrimental to health due to low contractile activity in large lower extremity muscle groups. This muscle inactivity can be measured with electromyography (EMG), but it is unknown how methodological factors affect responsiveness longitudinally. This study ranks 16 different EMG inactivity thresholds based on their responsiveness (absolute and standardized effect size, responsiveness) using data from a randomized controlled trial targeted at reducing and breaking up sedentary time (InPact, ISRCTN28668090). EMG inactivity duration and usual EMG inactivity bout duration (weighted median of bout lengths) were measured from large lower extremity muscle groups (quadriceps, hamstring) with EMG-sensing shorts. The results showed that the EMG inactivity threshold above signal baseline (3 μV) provided overall the best responsiveness indices. At baseline, EMG inactivity duration of 66.8 ± 9.6% was accumulated through 73.9 ± 36.0 s usual EMG inactivity bout duration, both of which were reduced following the intervention (−4.8 percentage points, −34.3 s). The proposed methodology can reduce variability in longitudinal designs and the detailed results can be used for sample size calculations. Reducing EMG inactivity duration and accumulating EMG inactivity in shorter bouts has a potential influence on muscle physiology and health.

## Introduction

Sedentary behaviour is defined as a seated or reclining posture with low energy expenditure^1^. Advancements in device-based monitoring of sedentary behaviour have enabled an accurate and fine-grained quantification of total sedentary behaviour and pattern of sedentary behaviour accumulation, both of which have shown to be detrimentally associated with health. A meta-analysis of randomized controlled trials concluded that reducing total sedentary time can modestly improve weight, waist circumference, percentage body fat, systolic blood pressure, insulin, and high-density lipoprotein cholesterol^2^. Observational evidence has further indicated that overall volume of sedentary time is associated with cardiovascular diseases and all-cause mortality^3,4^. Further, accumulating sitting time or sedentary time in a prolonged unbroken manner consistent with long periods of muscle inactivity is detrimentally associated with markers of cardiometabolic health, prevalent diabetes, incident cancer and all-cause mortality^5–9^. While high total sedentary time and a prolonged pattern of accumulation often co-exist, they may carry partly independent, and/or additive risk for health^5,6,9^. The 2020 physical activity guidelines now recommend reducing sedentary time for all adults, and especially for those with a low physical activity level^10^. However, there was insufficient evidence to make specific recommendations on the duration or frequency of bouts or breaks in sedentary behaviour^11^. One important step in advancing the field is to develop measurement methods that can capture the nature of sedentary behaviour according to the proposed mechanism of action i.e., the patterns how muscles are active/inactive during daily living^1^.

Lack of muscle activation is one of the key postulated mechanisms for the detrimental effects of sedentary time^12^. Muscles are inactive during prolonged periods of sitting resulting in declined contraction-mediated glucose uptake and suppressed blood-flow locally at the vicinity of motor units^12–17^. Severe muscle disuse, such as observed in bed rest studies, has been deleteriously associated with insulin resistance and impaired lipid trafficking and hyperlipidemia^18^. However, even shorter periods of prolonged sitting or lying time, such that is commonly observed in day-to-day activity^19^, have been associated with decreased insulin action in acute experimental studies^20^. When accumulated across several days, prolonged sitting can induce resistance to the benefits of physical activity bouts^21^. The importance of local muscle activation is illustrated in rodent and human studies where activation of muscle fibers results in improved insulin sensitivity without effect in the inactive contra-lateral leg^22,23^. Muscle insulin resistance can account up to 85–90% of the impairment in total body glucose disposal in both healthy and type 2 diabetic individuals^24,25^, illustrating the importance of local muscle activation for the systemic health benefits. However, only few studies have assessed muscle inactivity duration from large lower limb muscle groups in free-living conditions and less is known on the patterning of muscle inactivity bouts that are potentially relevant for health^26–28^.

Surface electromyography (EMG) is a method to assess muscle fiber electrical depolarizations and hyperpolarizations (i.e., muscle excitation) on the muscle fiber membrane, and therefore can be used to estimate muscle activity and inactivity periods. Identification of these electrically active, and electrically silent periods (EMG activity and inactivity bouts, respectively), can give insights on muscle inactivity patterns, which is likely one key pathway linking sedentary behaviour with health outcomes. One of the main limitations in this field is the lack of longitudinal studies that would allow the use of EMG inactivity to predict cardio-metabolic risk^12,29^. Before EMG inactivity can be reliably utilized in longitudinal studies to inform of health benefits, signal analysis requires systematic evaluation and sensitivity analysis. A number of analytical techniques to differentiate absence and presence of EMG signal onset have been evaluated in various study setups, with different electrodes and placements. While visual observation is reliable^30^, it is also more tedious than computerized analytical techniques that include setting the on–off threshold based on baseline deviation, amplitude distribution, or below a given proportion of a reference activity, like standing, walking, or maximal voluntary contraction-measured excitation^26–28,31–36^. While EMG measurement technology has been shown to be valid and reliable in measuring EMG amplitude^37^, the sensitivity of different thresholds to capture changes in EMG inactivity, i.e., responsiveness^38^, has not been systematically evaluated.

The aim of this study was to evaluate the responsiveness of EMG inactivity duration and pattern of accumulation, and compare these outcomes between different EMG inactivity thresholds using data from a randomized controlled trial. The trial targets were reducing and breaking up sedentary time, and therefore influence on the investigated EMG outcomes was expected.

## Materials and methods

Data for this study were collected in InPact project, which was a two-arm cluster-randomized controlled trial with the primary aim to reduce and break up sitting periods, and increase light intensity physical activity during work and non-work time^39–41^. Participants were healthy men and women having an occupation where they self-reportedly sat for more than 50% of their work time, and 3–8 year old children in all-day kindergarten or in the first grade of primary school. Cluster-sampling resulted in a total eligible sample of 71 intervention and 62 control participants^39^. The sample for the baseline laboratory study were a total of 86 intervention and control group participants having EMG data measured during laboratory sitting, standing and walking. Participants for the acute efficacy study were a total of 24 intervention and 24 control group participants based on having > 9 h artefact-free EMG data from two self-reportedly typical workdays (including work hours and non-work hours) from one day before, and one day within two weeks after the intervention^32^. The study was approved by the ethics committee of the Central Hospital, District of Central Finland, on March 25, 2011 (Dnro 6U/2011), and the participants signed an informed consent before measurements. All research was performed in accordance with the Declaration of Helsinki.

### Protocol

The acute efficacy of the InPact intervention was evaluated with EMG data measured during one typical workday before and after the intervention. Both days were preceded by a structured laboratory test protocol. For the first laboratory measurement, in the morning the participants’ height, weight, waist circumference, and total and regional fat mass were measured by dual-energy X-ray absorptiometry (LUNAR Prodigy, GE Healthcare, Chicago, Ill., USA) in a fasted state. Subsequently, a pair of snuggly fitting EMG shorts (Myontec Ltd., Kuopio, Finland) were worn and the laboratory activity measurements started. Sitting was measured while participants were sitting in front of a table where breakfast was provided and they were informed by the researcher about the diaries and questionnaires to be completed. In the beginning of the sitting period, participants were asked to sit still for five minutes, which was analysed as a silent sitting period. The expected duration of sitting period was 30 min, but this varied e.g., in case the participant had to hurry for work, or if the participant requested more information about the questionnaires, etc. After the sitting period, participants were asked to walk on a treadmill (OJK-1; Telineyhtymä, Kotka, Finland) at five, six and seven km/h (one minute each), and the five km/h load was used for further analyses. Standing was measured by asking participants to stand still, supporting weight on both legs (15 s). Finally, participants performed bilateral isometric maximal voluntary contraction (MVC) in a knee extension/flexion machine (David 220; David Health Solutions, Helsinki, Finland) with a 140° knee angle in both flexion and extension. After familiarization and warm-up, at least three 3- to 5-s maximal efforts with strong verbal encouragement were performed with a 1-min rest periods between trials. If torque improved by more than 5% in the last trial, more trials were performed. The laboratory activity measurements were repeated on the second measurement day in order to normalize the subsequent daily EMG data on the same signal MVC data.

After the laboratory activity measurements, the participants left for work and were expected to continue normal living while wearing the shorts until going to bed. Any abnormal tasks and behaviors, like having an unusually physically active work day, working overtime, being sick, etc., were to be reported, so that only structurally similar days were included for analysis. After these baseline measurements, the intervention group received tailored counseling. The postintervention measurements were performed within 2 weeks of the counseling session.

*Intervention* consisted of a 30-min lecture about the health hazards of prolonged sitting and encouragement to incorporate small physical activities into everyday routines^32,39^. The lecture was followed by a face-to-face discussion with the researchers, where participants were encouraged to think of feasible ways to reduce total and long sitting periods, to increase nonexercise physical activity, and to increase family-based activities. The research provided additional ideas and activity suggestions for the participants with an aim to set small-step goals for work time and non-work time. The goals were written into a contract signed by both the researcher and the participant.

### EMG recordings

EMG was recorded using shorts equipped with textile EMG electrodes for measuring muscle activity from the quadriceps and the hamstrings bilaterally (Myontec Ltd, Kuopio, and Suunto Ltd, Vantaa, Finland). The electrode sizes were 2.5 × 9.5–14 cm for quadriceps muscles, 1.5 × 7.5–8 cm for hamstrings muscles and 2.0 × 29–33 cm for lateral grounding electrodes, depending on the size of the shorts. Due to the size of the electrodes, the EMG shorts capture signal from a larger area compared to typical bipolar electrodes, and the data can be considered to represent activity and inactivity of muscle groups^31,42^.

### Data analysis

#### EMG signal

**Figure 1:**
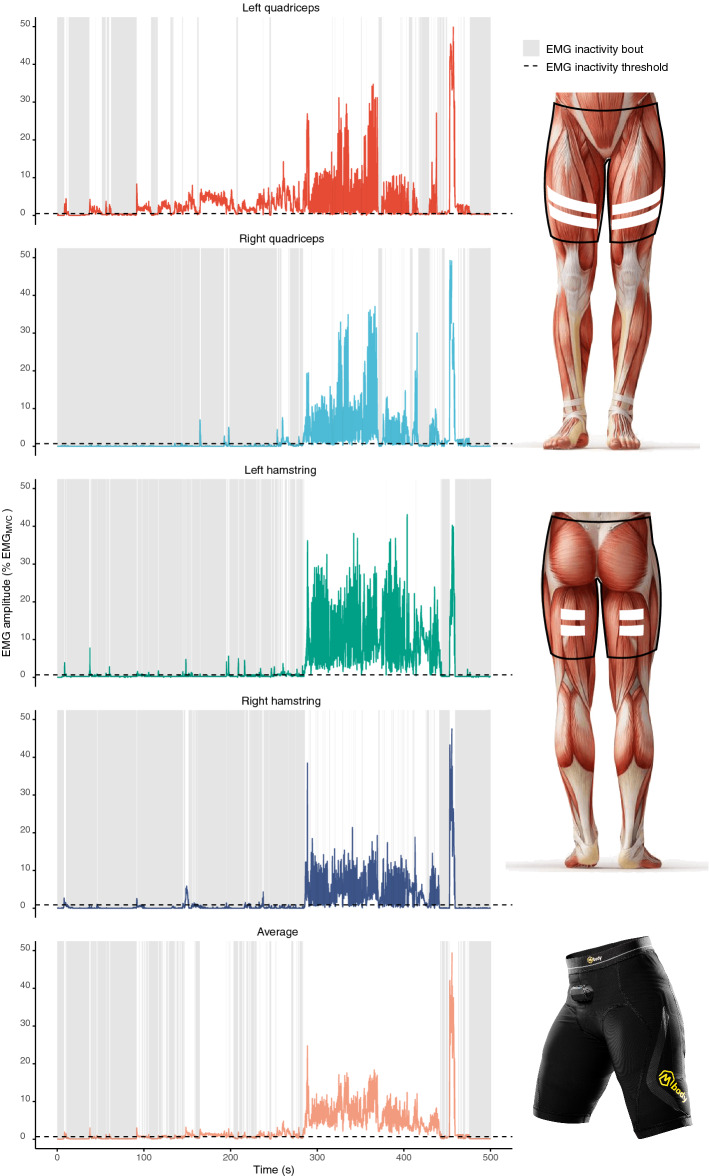
Example timeline of normalized, smoothed, baseline corrected EMG data for left and right quadriceps and hamstring muscle groups as well as for averaged channel. EMG inactivity bout was defined as a period (grey areas) when EMG amplitude was below the EMG inactivity threshold (dashed line above baseline). EMG shorts electrode placement above quadriceps and hamstring muscle groups is shown on right. Image downloaded from Adobe Stock under Education license. Picture of EMG shorts published with a permission from Myontec Ltd.

EMG data analysis details are presented in Supplemental material. EMG signal was logged on a module attached on the shorts’ waistline (Fig. 1) in its raw form with a sampling frequency of 1000 Hz and band-bass filter of 50–200 Hz (−3 dB). The raw EMG signal was first rectified and the root mean squared (RMS) value of each of the four channels was calculated over non-overlapping 100 ms (10 Hz, old model) or 40 ms epochs (25 Hz, new model, details in Supplemental material).

#### Signal analysis

EMG signal analysis was performed in RStudio Version 1.3.1093 (RStudio, PBC).

Each channel was normalized to the respective EMG_MVC_ value measured during the isometric maximal voluntary contraction, yielding %EMG_MVC_. The repetition with the highest force level was chosen, from which the most consistent 1-s mean EMG amplitude was used for each channel. EMG signal is random in nature because the set of recruited motor units constantly changes below the electrode measurement area, resulting in an arbitrarily superposed amplitude measured by the electrodes^43^. The degree of non-reproducibility of the signal can be minimized by applying a smoothing algorithm, which cuts any steep spikes from the signal, and produces a “linear envelope”. This was done by applying a moving average algorithm on each channel. An epoch length between 20 ms (rapid activities) and 500 ms (slow movements) has been previously recommended^43^. Moreover, it has been previously suggested that a ~ 200 ms window would be reasonable for concluding whether a muscle is on or off^29,44^. In thepresent study, a 200 ms moving average epoch was utilized. EMG signal baseline can sometimes shift due to improper contact between the skin and the electrodes. The potential shift was minimized by applying a 5-min moving window, which searched for the minimum value from this window and subtracted this from the value preceding the window. Baseline correction improves data normality as presented in Supplemental Fig. S2 online. In cases of improper functioning of the measurement device, or imbedance problems between skin and electrodes due to loose contact, the signal may contain artefacts. These artefacts were visually screened by plotting the data in R Studio, and the corresponfing channels were removed as previously described^27^. The effect of channel removal has been previously evaluated and further details are presented in Supplemental Figs. S3 and S4^39^. In order to evaluate overall thigh muscle region EMG inactivity periods, the normalized, smoothed, baseline corrected channels from the left and right quadriceps and hamstring muscles were averaged (Fig. 1).

### EMG inactivity thresholds

Several threshold options were tested based on previous research, and practical and analytical considerations.

*Relative to maximal voluntary contraction-thresholds (%EMG*_*MVC*_*)* have been previously employed in several studies^30,34–36,45^. In thepresent study, four different thresholds were considered (*1, 2, 3, 4%*).

*Absolute thresholds (Absolute)* were set above the signal baseline. The rationale for testing absolute threshold is that it can be set close to signal baseline regardless of individual capacity, since muscle electrical on/off behavior is not dependent on capacity. Therefore, we considered this threshold to be potentially sensitive to distinguish EMG inactivity and activity periods. It is important to note that the signal and the threshold were normalized to the maximal voluntary contraction, such that the difference between signal baseline and the threshold remained the same in absolute terms (e.g., if EMG_MVC_ for a given channel was 120 μV, the 3 μV threshold was 3 μV/120 μV = 2.5%EMG_MVC_). Four different absolute thresholds were considered (1, 2, 3, 4 μV).

*Standing-based thresholds (Standing)* have been used in previous research quantifying EMG-shorts measured muscle inactivity during daily living^26,27,32,46^. The rationale for standing-based thresholds is that standing is a common low-intensity upright activity type that is not considered as sedentary behaviour^1^. Therefore, mean muscle EMG during standing can be considered as a cutpoint to differentiate sedentary behaviour from physical activity. Four different fractions below mean standing EMG were considered (*0.6x, 0.7x, 0.8x, 0.9x*).

*Baseline standard deviation thresholds (Baseline sd).* The rationale for using baseline deviation as the threshold is based on the fact that sEMG baseline is rarely silent, and a threshold that is based on the statistical deviation of baseline can correct for the erratic changes in baseline activity^30^. In the present study, four Baseline sd thresholds (*1sd, 2sd, 3sd, 4sd*) were calculated based on multiples of standard deviation measured during the laboratory silent sitting period.

#### EMG inactivity outcomes

*EMG inactivity duration* was analysed as the summed duration when the EMG amplitude was below the EMG inactivity threshold and is presented as a proportion of file length (*EMG inactivity duration (% of measurement time),* Fig. 1).

*Usual EMG inactivity bout duration* is a weighted median of EMG inactivity bout lengths (Fig. 1)*.* EMG inactivity bout durations are positively skewed, and typical summary statistics can not be used to robustly characterize the data. As suggested by Chastin and Granat^47^, we used a non-linear regression technique (Levenberg-Marquart) to fit a sigmoid function $\frac{{t}^{n}}{({t}^{n}+ {{W}_{50\%}}^{n})}$, where *t* is the EMG inactivity bout duration, *n* a free parameter, and *W*_*50%*_ the usual EMG inactivity bout duration, above or below which 50% of EMG inactivity duration is accumulated. Details for this calculation are presented in the Supplemental material.

### Statistical analyses and responsiveness indices

EMG inactivity duration measured with different thresholds was compared within the laboratory activities in order to provide an overview of results within activities considered as sedentary (sitting) and active (standing and walking). Spearman correlation coefficients between EMG inactivity duration and total fat percentage as well as leg fat mass were calculated to evaluate whether leg fat mass or total body composition are potential confounders in EMG inactivity analyses^48^. Median and interquartile range of EMG inactivity duration and usual EMG inactivity bout duration at baseline were plotted to visually compare outcomes between the thresholds.

Responsiveness is defined as the ability of an instrument to detect change accurately, but can be operationalized in a myriad of ways^38^. We selected metrics that consider both a within-individual change, between-group change, as well as variability in both. While within-individual change is considered relevant for responsiveness, between-group change is important in the context of randomized controlled trials, including also a control group^38^. The reported outcomes can also be used to estimate sample sizes in both parallel and cross-over intervention designs (details provided in the Supplemental material). The main aim is to compare which thresholds provide the best responsiveness overall considering the both outcomes and responsiveness indices.

*Absolute effect size* was calculated as the difference in within intervention and control group changes between follow-up and baseline measurements according to Eq. 1.

1 $$Absolute\, effect\, size \,\left({ES}_{Abs}\right)= {\Delta X}_{Int}- {\Delta X}_{Cont}$$

*Standardized effects size,* also referred to as sensitivity to change, or Cohen’s d, was used to assess whether the observed change is larger than the pooled variability and is calculated as the mean change within the intervention group, divided by the intervention group pre and post pooled standard deviation as described in Eq. 2. The sensitivity to change increases, if the variability decreases, and/or if the within group mean change between baseline and follow-up measurements increases.

2 $$Standardized\, effect\, size= \frac{{\Delta X}_{Int}}{\sqrt{\frac{{SD({X}_{Int1})}^{2}+{SD({X}_{Int2})}^{2}}{2}}}$$

*Responsiveness*, as proposed by Guyatt et al. 1987, can be calculated by taking the ratio of clinically important difference to the variability in stable (control) subjects^49^. While clinically important difference for EMG inactivity duration and usual EMG inactivity bout duration will depend on the health outcome and the setting where used, we use the absolute effect size since it is known for the present intervention. Responsiveness was calculated based on Eq. 3 as the ratio of absolute effect size to the within-individual standard deviation within control group. In Eq. 3, X_Con1_ and X_Con2_ are baseline and follow-up measurements within control group, and n is number of observations, i.e. the number of X_Con1_ and X_Con2_ differences.

3 $$Responsiveness= \frac{{ES}_{Abs}}{\sqrt{\frac{\sum {({X}_{Con2}-{x}_{Con1})}^{2}}{2n}}}$$

The rank ordering of different responsiveness indices was compared with Friedman’s test. All analyses were performed in RStudio Version 1.3.1093 (RStudio, PBC).

## Results

### EMG inactivity during laboratory sitting, standing and walking

**Figure 2:**
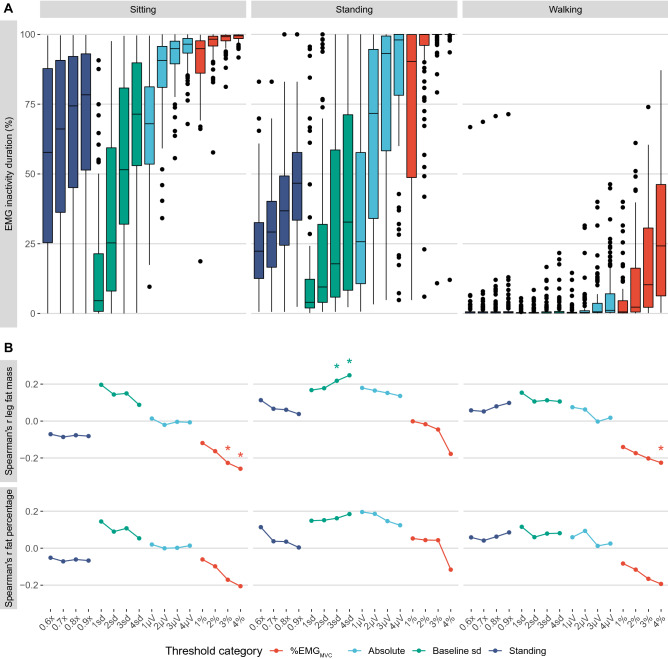
EMG inactivity duration (**A**) and Spearman correlation coefficients (**B**) between EMG inactivity duration and leg fat mass and total fat percentage across EMG inactivity threshold categories (on x-axis) within laboratory sitting, standing and walking. Asterix denote to significant correlations (*P* < .05).

Laboratory sample participants consisted of 46 women and 40 men, aged 37.7 ± 4.98 years (range 29–50 years), having a BMI of 24.4 ± 3.46 kg/m^2^ (range 18.3–35.6 kg/m^2^), leg fat mass of 9.44 ± 3.64 kg (range 2.54–19.5 kg), and body fat percentage of 39.2 ± 13.5% (range 11.4–66.9%), on average. Sitting was measured for 26.2 ± 14.4 min, standing still for 0.34 ± 0.16 min, and walking on a treadmill at 5 km/h for 0.98 ± 0.43 min. Figure 2A shows that EMG inactivity duration during sitting in laboratory ranged from 4.6% to 99.5% of measurement duration between different EMG inactivity thresholds. During standing in the laboratory, EMG inactivity duration ranged from 4.0 to 100.0%. During walking at 5 km/h on treadmill, all thresholds captured a low EMG inactivity duration due to cyclic nature of gait (median 0.2–24.2% of measurement duration, Fig. 2A).

Figure 2B illustrates that all correlations between EMG inactivity duration and leg fat mass, and fat percentage, were weak (−0.3 < r < 0.3). However, leg fat mass correlated negatively with EMG inactivity duration measured with %EMG_MVC_ thresholds during sitting (3% r = −0.23, 4% r = −0.26, *p* < 0.05) and walking (4% r = −0.23, *p* < 0.05), and positively with EMG inactivity duration measured with baseline sd threshold during standing (3sd r = 0.22, 4sd r = 0.25, *p* < 0.05). Overall, body composition or leg fat mass were not confounding factors in EMG inactivity duration analysis, particularly with absolute and standing -based thresholds, or with any low threshold.

### Daily EMG inactivity duration and accumulation at baseline

**Figure 3:**
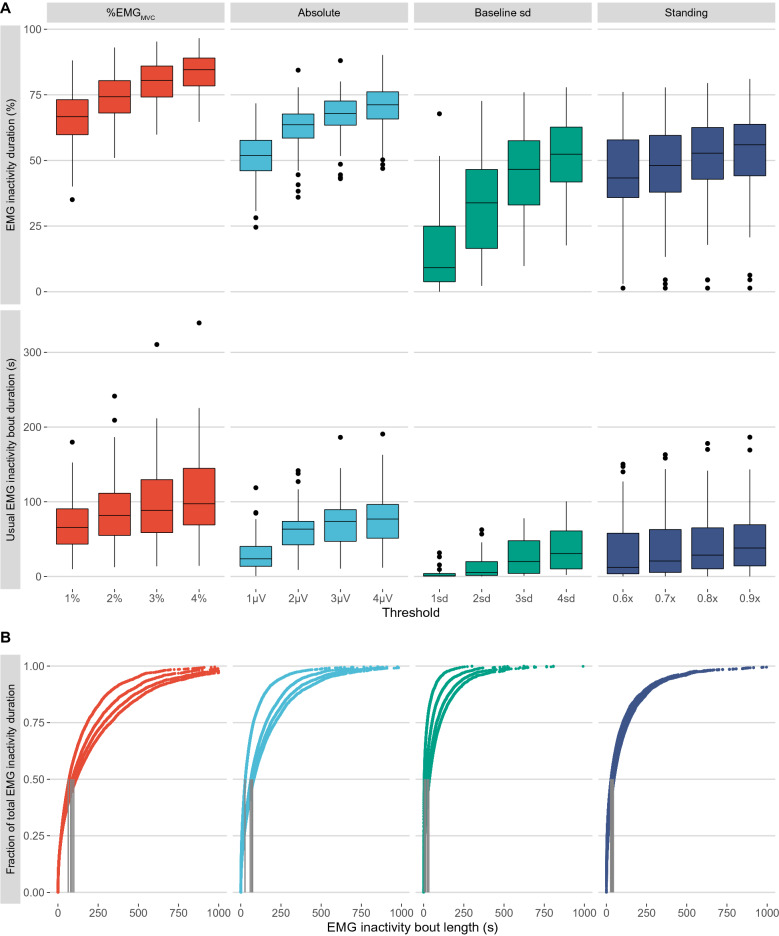
(**A**) Baseline EMG inactivity duration and usual EMG inactivity bout duration across different thresholds. B) EMG inactivity bout accumulation patterns including data from all participants. Usual EMG inactivity bout duration was estimated individually as an EMG inactivity bout length above and below which 50% of total EMG inactivity duration is accumulated, as illustrated by the vertical grey lines.

A total of 24 intervention (women n = 15) and 24 control (women n = 13) group participants had 11.8 ± 1.1 h data from daily measurements at baseline and during follow-up. At baseline, EMG inactivity duration and usual EMG bout duration increased consistently when the threshold increased, across all threshold categories, both outcomes being highest with a *4% EMG*_*MVC*_ threshold and lowest with a *Baseline 1sd* threshold (Fig. 3A). Figure 3B illustrates accumulation patterns of EMG inactivity duration. The lower the threshold, the larger fraction of total EMG inactivity duration consisted of short EMG inactivity bouts, resulting in a shorter usual EMG inactivity bout duration. This illustrates that some of the very short bouts had a low amplitude, and therefore they were not captured with the higher thresholds.

### Responsiveness

Absolute effect size, standardized effect size and responsiveness were calculated and ranked between the thresholds, and are presented in Table 1. Rankings varied depending on the responsiveness indice and outcome in question. For EMG inactivity duration, absolute effect size and responsiveness were largest with baseline sd and standing thresholds. Standardized effect size was largest with absolute thresholds, whereas baseline sd thresholds were among those showing the lowest standardized effect size. For usual EMG inactivity bout duration, all responsiveness indices were largest with %EMG_MVC_ and absolute thresholds (Table 1).

**Table 1:** Rank ordering of responsiveness indices. For EMG inactivity duration, absolute effect size was measured in percentage points (pp), where follow-up EMG inactivity duration (% of measurement duration) was subtracted from that at baseline. For Usual EMG inactivity bout duration, absolute effect size was measured in seconds (s).

Rank	EMG inactivity duration						Usual EMG inactivity bout duration						Overall sum of ranks	
	Absolute effect size (pp)		Standardized effect size (unitless)		Responsiveness (unitless)		Absolute effect size (s)		Standardized effect size (unitless)		Responsiveness (unitless)			
1	3sd	−10.7	2 μV	−0.49	0.9x	−1.91	4%	−39.6	4 μV	−0.78	3 μV	−1.18	3 μV	33
2	0.7x	−10.4	1 μV	−0.43	0.8x	−1.83	3%	−38.9	3 μV	−0.75	4 μV	−1.17	2 μV	34
3	0.8x	−10.3	3 μV	−0.41	0.7x	−1.40	2%	−37.3	2%	−0.71	1%	−1.09	4 μV	35
4	0.9x	−10.3	4 μV	−0.38	3sd	−1.16	4 μV	−36.4	2 μV	−0.68	2 μV	−1.03	0.9x	39
5	2sd	−10.0	1%	−0.37	0.6x	−1.15	3 μV	−34.3	1%	−0.66	2%	−0.99	1%	39
6	1 μV	−9.7	0.9x	−0.36	4sd	−1.15	1%	−31.4	3%	−0.64	3%	−0.89	1 μV	42
7	4sd	-9.5	0.8x	–0.34	2sd	-0.95	2 μV	-27.4	4%	-0.52	4%	-0.80	0.8x	45
8	0.6x	−8.1	4sd	−0.29	1 μV	−0.94	1 μV	−18.8	0.9x	−0.27	4sd	−0.71	2%	47
9	2 μV	−6.0	2%	−0.29	2 μV	−0.91	0.9x	−15.2	1 μV	−0.25	1 μV	−0.69	4sd	50
10	1%	−5.0	3sd	−0.27	1%	−0.83	0.8x	−12.7	4sd	−0.24	3sd	−0.50	3sd	52
11	3 μV	−4.8	3%	−0.26	3 μV	−0.82	4sd	−11.2	0.8x	−0.18	0.9x	−0.48	3%	54
12	4 μV	−4.2	0.7x	−0.24	4 μV	−0.76	0.7x	−10.8	0.7x	−0.08	0.8x	−0.39	0.7x	54
13	1sd	−3.8	4%	−0.23	2%	−0.65	0.6x	−9.8	3sd	−0.08	0.7x	−0.33	4%	59
14	2%	−3.5	0.6x	−0.20	3%	−0.52	3sd	−6.3	0.6x	−0.05	0.6x	−0.31	0.6x	68
15	3%	−2.5	2sd	−0.15	4%	−0.41	2sd	−2.3	2sd	0.01	1sd	−0.28	2sd	73
16	4%	−1.8	1sd	−0.04	1sd	−0.40	1sd	−0.7	1sd	0.03	2sd	−0.23	1sd	92

When considering the ranks of all responsiveness indices and both outcomes, absolute threshold 3 μV had the best responsiveness to measure changes in EMG inactivity duration and usual EMG inactivity bout duration (Table 1).

## Discussion

Muscle inactivity is one of the mechanisms in the metabolic risks of sedentary behaviour, yet only a few studies have measured longitudital changes in this outcome^12,32,50^. The present study evaluated the methodological aspects with a view to improve responsiveness of EMG inactivity measurement, which, in addition to validity and reliability, is a key element in measuring change over time^29,38,49^. The present results showed that methodological choices had a considerable influence on the EMG inactivity duration, pattern of accumulation, and the responsiveness indices investigated. With an EMG inactivity threshold set above signal baseline changes in EMG inactivity duration and pattern of accumulation can be measured with an acceptable sensitivity and responsiveness. The proposed methodology can reduce variability and sample size requirements in longitudinal EMG studies.

The present results show that methodological factors have a significant influence on EMG inactivity duration. Any differences in EMG inactivity duration may be due to baseline noise, or physiologically relevant activity. Therefore, EMG inactivity duration was first compared between thresholds within sitting, standing and walking, which are typically sedentary, light-intensity, and moderate intensity activities, and are expected to differ in their EMG inactivity duration due to physiological activity. Even though sitting is considered a sedentary behaviour, some EMG activity was measured during sitting with all of the thresholds. This is consistent with previous research showing heterogeneity in how people activate their muscles during sitting and standing due to changing posture or fidgeting^17,51,52^. On average, standing is more active (mean amplitude 2.2% EMG_MVC_) than sitting (1.0%EMG_MVC_) due to postural muscle activation, and is expected to reduce thigh muscle inactivity compared to sitting^26^. However, many of the compared thresholds (like 2–4% EMG_MVC_) captured a high EMG inactivity duration (> 92%) during both sitting and standing. Physiological characteristics can also play a role in standing muscle activity, because overweight people need to carry a higher body mass during standing^17^. Body composition, and particularly fat tissue thickness below the recording electrodes, can also reduce EMG amplitude and confound EMG inactivity analysis^48^. The correlations between EMG inactivity duration and body fat were however low within all of the thresholds, particularly with absolute and standing -based thresholds. The thresholds that were individually based on standing mean amplitude, and absolute thresholds that were set above signal baseline (particularly at 1 and 2 uV), also provided highest differences in EMG inactivity duration between sitting and standing.

The dataset from RCT targeting decreasing and breaking up sedentary time was used to compare which thresholds provide the best responsiveness in EMG inactivity duration and pattern of accumulation. Pursuing these behavioural targets is expected to decrease EMG inactivity duration and shift pattern of accumulation towards shorter EMG inactivity bouts^32^. The baseline results showed that EMG inactivity duration decreased curvilinearly when the threshold increased, which is consistent with a previous study measuring 24-h EMG activity from quadriceps femoris muscle^28^. Responsiveness indices showed that absolute effect size, responsiveness and standardized effect size for reduction of EMG inactivity duration were better at lower thresholds within absolute and %EMG_MVC_ thresholds (e.g., better responsiveness indices with 1 μV compared to 4 μV threshold). Such a pattern was less obvious within baseline sd and standing thresholds, where the better responsiveness indices were reached with higher rather than lower thresholds. A potential explanation could be in the capability of different thresholds to measure difference in EMG inactivity between sitting and standing, since standing is a common replacement activity for sitting and was one of the intervention’s behavioural targets^32^. For %EMG_MVC_ and absolute thresholds, the difference in sitting and standing EMG inactivity duration increased at lower thresholds, whereas the opposite was true for baseline sd thresholds. For standing-based thresholds, the difference was stable across the thresholds, which is understandable given standing itself was used as the threshold criteria.

For usual EMG inactivity bout duration, all responsiveness indices were generally greater at higher thresholds. A potential reason is that lower thresholds capture more very brief EMG inactivity bouts (e.g. usual EMG inactivity bout for 1 μV threshold was 23.6 s at baseline), and further manipulating such fast-paced pattern is behaviourally irrelevant and impossible, yet at higher thresholds the usual EMG inactivity bouts correspond more to actual behaviour change. Given different rankings between thresholds for the outcomes, investigators can also consider selecting the threshold depending whether their primary outcome is total EMG inactivity duration (where a lower threshold provides better responsiveness) or usual EMG inactivity bout duration (where a higher threshold provides better responsiveness). When considering both outcomes and all responsiveness indices, absolute threshold 3 μV provided the best responsiveness to detect changes in EMG inactivity duration and usual EMG inactivity bout duration.

The EMG inactivity outcomes investigated are likely physiologically relevant and their associations with health outcomes should be further evaluated. Total EMG inactivity duration has been shown to be adversely associated with HDL cholesterol and triglycerides in a sample of physically active adults^26^. In addition, the pattern how this total volume of EMG inactivity is accumulated can be relevant for health. An analysis of the accumulation pattern showed that EMG inactivity duration was accumulated through shorter EMG inactivity bouts, the lower the EMG inactivity threshold was (median 1–97 s). Accelerometer-measured usual sedentary bout duration has been reported to be around 17–26 min in healthy participants, and several studies have reported that accruing total sedentary time in longer sedentary bouts is adversely associated with health^5–9,47^. However, in one study short sedentary bouts were found to be detrimental^53^. Most of the accelerometer-measured sedentary bouts are likely actual sitting bouts. Given that EMG inactivity bouts are considerably shorter, usual EMG inactivity bout likely does not directly correspond to sitting. Instead, EMG inactivity bout can be interrupted by any muscle activity, like changing posture or fidgeting while seated^17^. Fidgeting-like activities within prolonged sitting have been shown to improve limb blood flow and postprandial glycemic control in people with obesity^52^. Already a very low level of voluntary muscle excitation, or involuntary excitation invoked by electrical stimulation, can increase insulin sensitivity in inactive patients or during bed rest^54,55^. In experimental laboratory studies, decreasing the overall sedentary duration, or increasing frequency of activity breaks, has beneficially affected glucose sensitivity, despite the matched total energy expenditure between the protocols^56,57^. Therefore, EMG inactivity duration and usual EMG inactivity bout duration can be metabolically relevant outcomes and their influence on health outcomes should be investigated in longitudinal studies.

EMG shorts provide similar information than traditional bipolar EMG electrodes on the EMG signal amplitude but with smaller day-to-day coefficient of variation^37^. EMG shorts have a relatively large electrode area and a longer inter-electrode distance, and they measure the electrical activity, or lack of thereof, several muscle fibers. Therefore, the present data cannot be directly compared to typical bipolar electrode measurements, which captures signal from a smaller area. It was recently shown that ankle muscles do not activate homogeneously during standing. Some muscles, or some parts of individual muscles, may be “silent” during the excitation of others^58^. Therefore, an electrode with a small pick-up area may not be representative of the whole muscle at a given timepoint. A longer inter-electrode distance increases the underlying pick-up volume, and thus improves how representative the signal is of the whole muscle excitation^59^. One argument to support the shorter inter-electrode distances (such as the typically used 2 cm) is to avoid cross-talk, that is signal emanating from adjacent or deep muscle fibers, which interferes with the signal of interest from the specific electrode placement^60^. The specific placement of electrodes may decrease the reproducibility of signal especially in longitudinal designs, because electrodes should be placed in the exact same positions at the follow-up measurements. The signal collected with EMG shorts is not intended to be selective of specific muscle fibers, yet is representative of several muscle fibers and covers some parts of the adjacent muscles (Fig. 1). This fact, combined with the larger inter-electrode distance, may explain the better reproducibility of signal as compared to bipolar electrodes^37^. On the other hand, the large textile electrodes reduce the high-frequency component in the signal, and while the high pass filter corner frequency of 50 Hz can effectively reduce movement artefact during rapid movements, it may also cut some of the signal originating from muscles^61,62^.

This study has some limitations. The responsiveness is specific to the populations and interventions tested^38,63^, and these results only describe responsiveness indices in a relatively small sample of healthy, normal weight participants employed in desk-based occupations, and may not be generalizable to other populations or interventions. Due to the demanding nature of EMG measurement, only one day before and after intervention was measured. For accelerometer studies a minimum of 3–5 days of data have been proposed to characterize daily physical activity with good reliability^64^. To improve reliability, we asked participants to select days that are similar in their work tasks and leisure activities. However, this can decrease the generalizability of the findings and participants may have picked days that enable more activity than would be possible over longer time frames.

In conclusion, surface electromyography has been widely used to investigate why and how physiological adaptations occur at the muscle level, and muscle inactivity has been hypothesized to be a key mechanism in metabolic risks of prolonged sitting. However, there is little longitudinal work considering changes in EMG inactivity duration and pattern of accumulation during normal daily life. The present results show that methodological factors have a significant impact on responsiveness of EMG inactivity duration and pattern of accumulation. An absolute threshold set above signal baseline (3 μV) provided overall the best responsiveness when considering both of these outcomes. The proposed methods decrease variability in longitudinal EMG data and reduce sample size requirements. Furthermore, we report within and between individual standard deviations and pre-post correlations for all thresholds in Supplemental material that can be used for sample size calculation. Future studies should test how changes in EMG inactivity duration and pattern of accumulation change metabolic health.

## Supplementary Information

Supplementary Information.

## Data Availability

Upon reasonable request to Arto J. Pesola, data supporting the conclusions of this manuscript will be made available.
