# Supplementary material for: Responsiveness of electromyographically assessed skeletal muscle inactivity: methodological exploration and implications for health benefits
Source: Sci Rep. 2022 Dec 2;12:20867. doi: 10.1038/s41598-022-25128-y (PMC9718848; doi:10.1038/s41598-022-25128-y)
Supplement: Supplementary file 1 — Supplementary Information. [file 41598_2022_25128_MOESM1_ESM.pdf]

Supplemental material: EMG shorts data analysis

In: Responsiveness of Electromyographically Assessed Skeletal Muscle Inactivity – methodological exploration and implications for health benefits

Pesola A., Gao Y, Finni T

This supplemental material gives further details on the EMG shorts data analyses. The first part covers details of signal analyses and presents data comparisons between different analytical decisions. The second part reports between and within-individual standard deviations and pre-post correlations that can be used for sample size calculation purposes.

EMG shorts signal analysis steps

1. **Comparison of 10 Hz and 25 Hz signals**
   In the older EMG shorts and modules, the RMS value was calculated at 10 Hz, and in the newer EMG shorts and modules at 25 Hz. Currently 25 Hz is the standard used, but both 10 Hz and 25 Hz data was used for this manuscript. Figure S1 shows that there were no large differences in EMG inactivity duration distribution density between the models, except within Standing thresholds the 10 Hz data distribution was more left skewed (more participants with a low EMG inactivity duration). Furthermore, usual EMG inactivity bout duration was positively skewed in 10 Hz data across all thresholds, suggesting that 10 Hz data is accumulated in longer usual bouts.

2. **Normalization**
   Each channel was normalized to channel-specific EMG_{MVC} measured during maximal voluntary contraction, as described in methods.

3. **Smoothing**
   A 200 ms moving average algorithm was applied on each channel. Figure S2A shows that using either the 200 ms, or alternatively 1000 ms, smoothing window length improved EMG inactivity duration distribution density normality within Absolute threshold 1 μV, Baseline sd and Standing thresholds (Figure S2A).

4. **Baseline correction**
   A 5-minute moving baseline correction filter was next applied on each channel, which searched for the minimum value from this window and subtracted this value from the value preceding the window. As presented in Figure S2B, using either the 5 minute, or 30 minute, baseline correction window considerably improved data normality as compared to not correcting baseline.

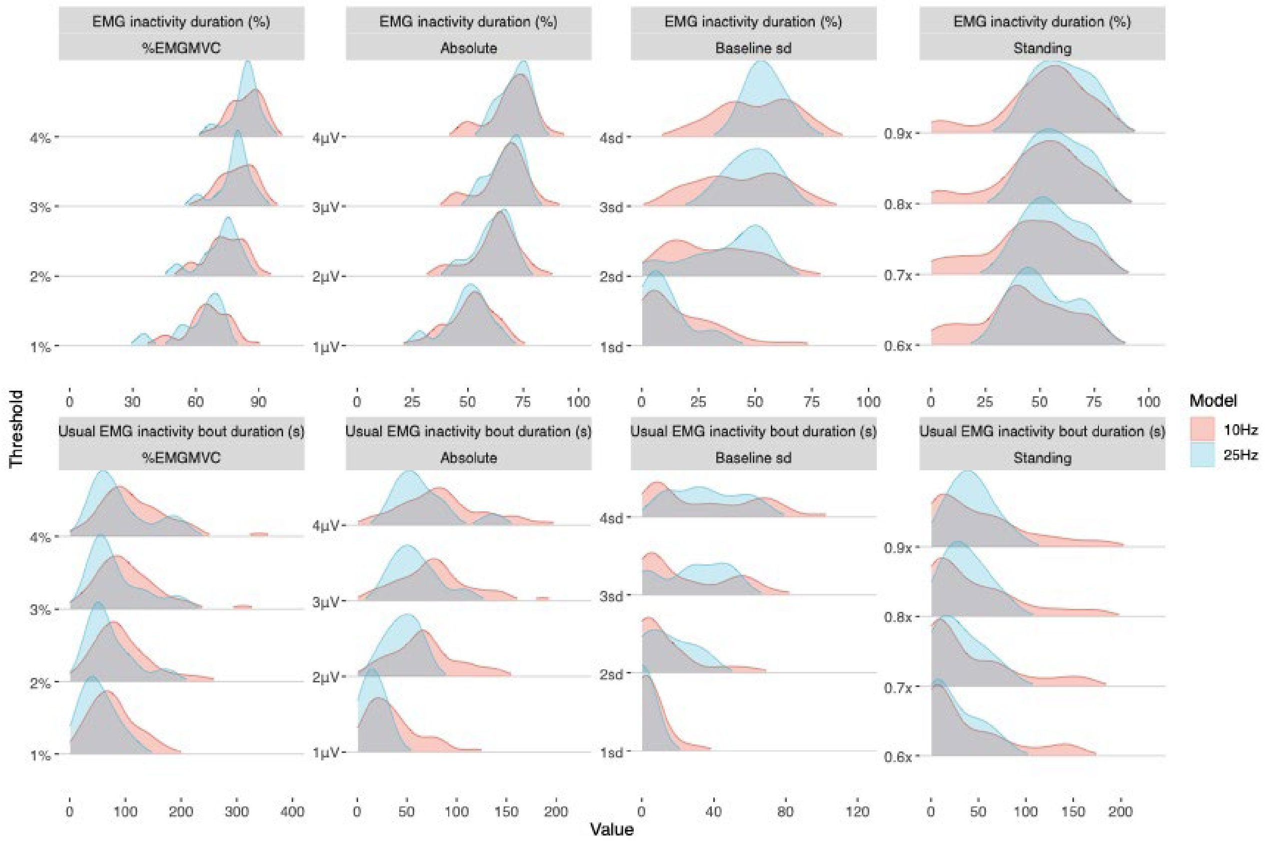

Figure S1. Distribution density of EMG inactivity duration and Usual EMG inactivity bout duration between data measured with 10 Hz and 25 Hz models.

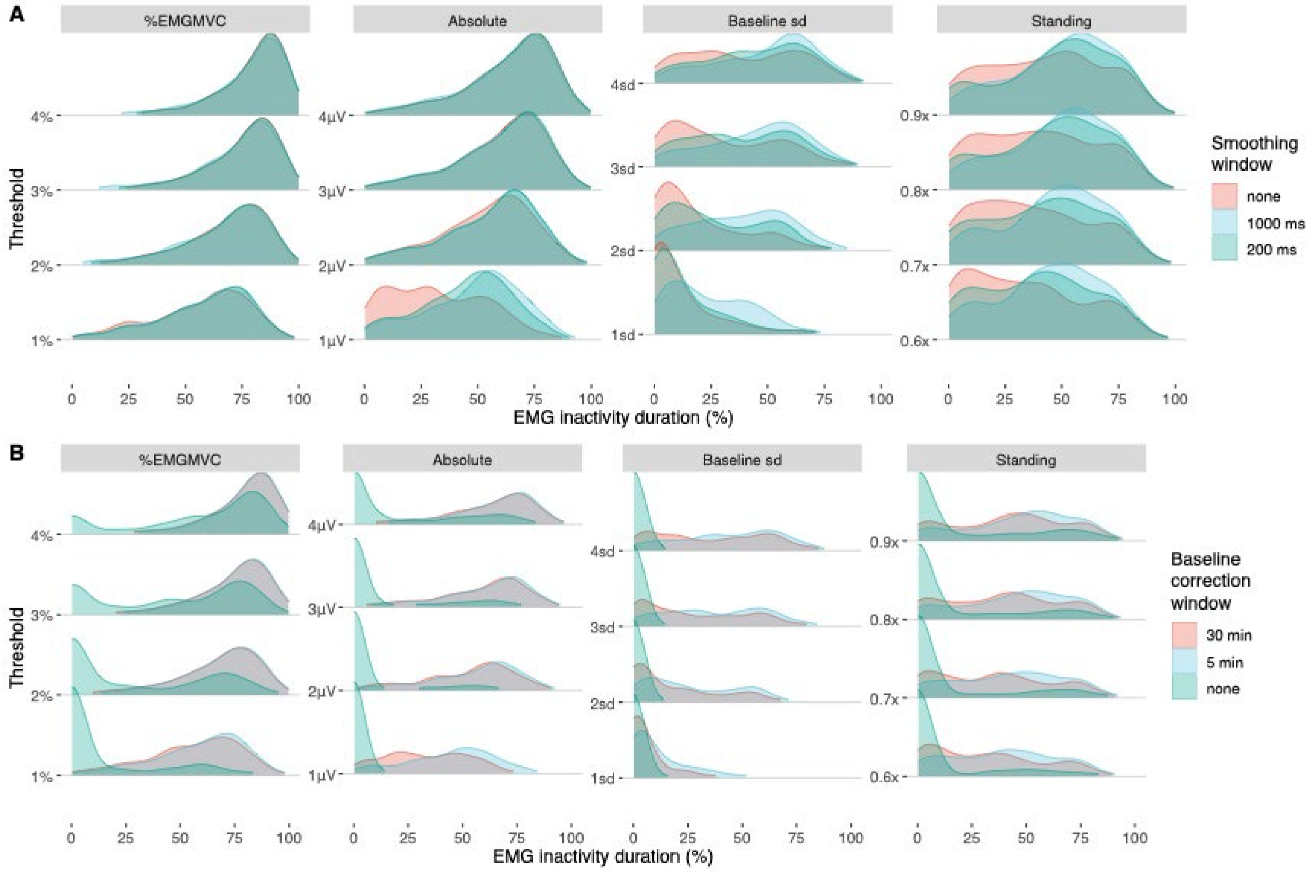

Figure S2. Distribution density of EMG inactivity duration analyzed with different A) smoothing window lengths and B) baseline correction window lengths.

5. **Artefact removal**

EMG signal may contain artefacts due to improper fitting of the shorts, or movement of the electrodes in relation to skin, causing artefacts that can be visually detected from the signal. Typical cases include a constantly very high amplitude, or a considerably drifting baseline, which cannot be corrected for by the baseline correction filter. Such artifacts were visually screened and in cases artefact was prolonged (>30 min), the corresponding channel was removed. To sustain longitudinal comparability, the corresponding channels were also removed from the other measurement point. Figure S3 shows EMG inactivity duration and Usual EMG inactivity bout duration at baseline between participants having different number of channels included. The distribution density of EMG inactivity duration was similar between participants regardless of channel number. However, there were some differences in Usual EMG inactivity bout duration distribution density, particularly within Standing thresholds. The small differences suggest that thigh muscles activate in a synergistic manner, and if any of the measured muscle groups is active (or inactive), other thigh muscle groups likely behave in a related manner.

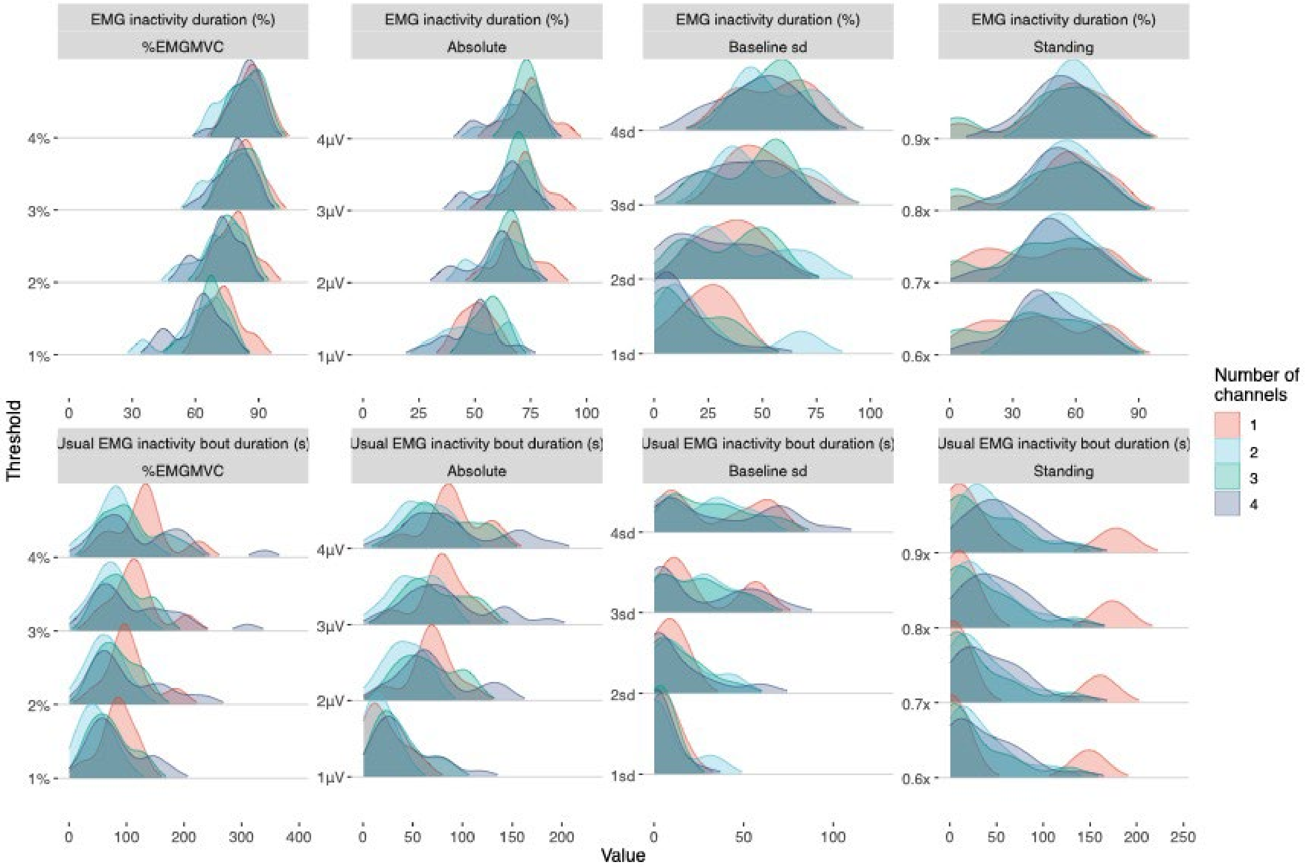

Figure S3. Distribution density of EMG inactivity duration and Usual EMG inactivity bout duration between participants having different number of channels.

6. **Averaging**

The normalized, smoothed, baseline corrected, and artefact free channels were finally averaged to represent overall thigh muscle region EMG inactivity. Figure S4 presents distribution densities for EMG inactivity duration and usual EMG inactivity bout duration calculated from averaged channels including different combinations of hamstring and quadriceps muscle groups. Channel combinations had little differences in EMG inactivity duration within other thresholds, except within Standing thresholds where quadriceps only data had a lower EMG inactivity duration. A similar tendency was seen for usual EMG inactivity bout duration within Standing thresholds, where quadriceps only data had a shorter, and hamstrings only data had longer, usual EMG inactivity bout duration.

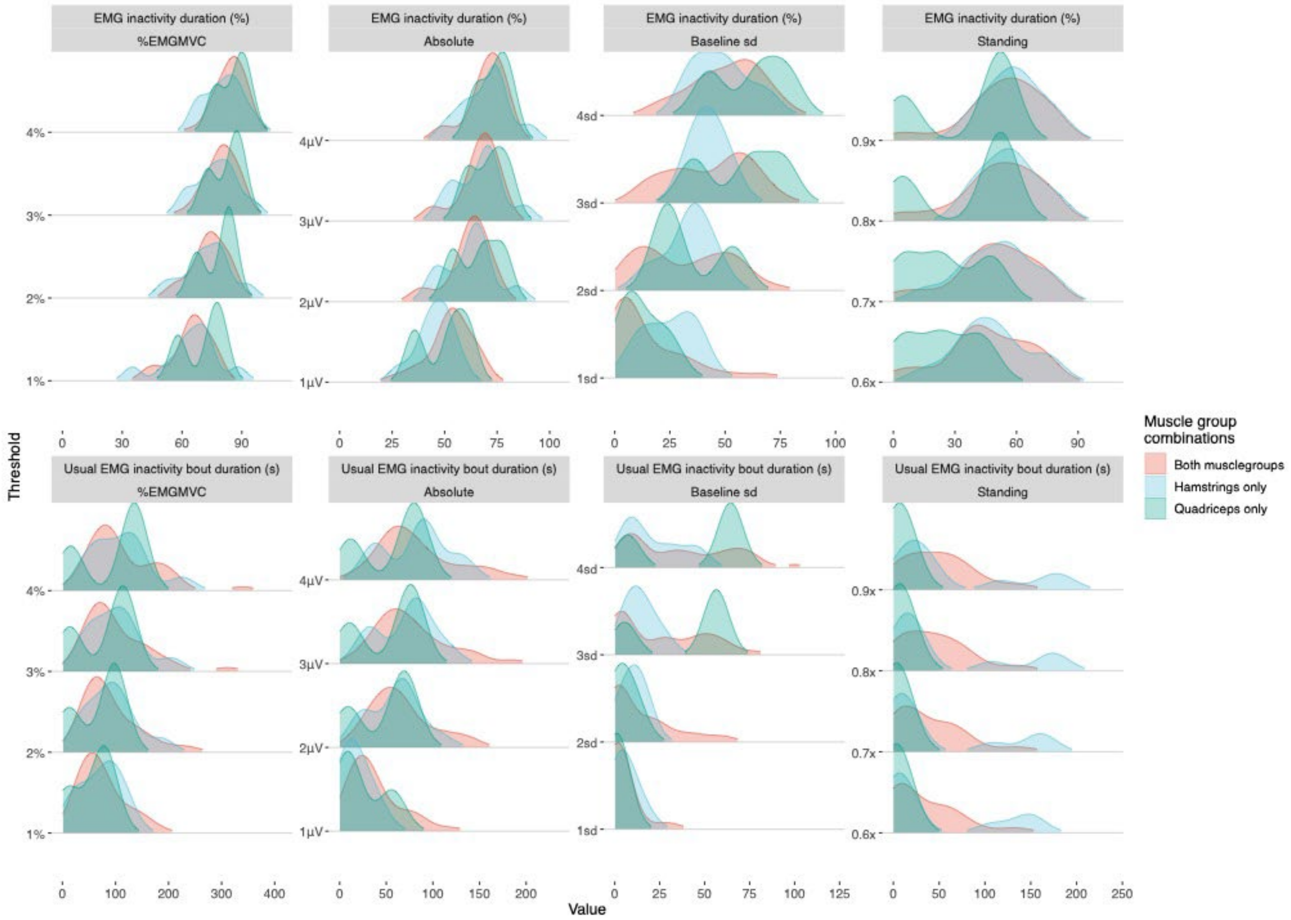

Figure S4. Distribution density of EMG inactivity duration and Usual EMG inactivity bout duration between participants with averaged data calculated from different muscle group combinations.

7. **Usual EMG inactivity bout duration calculation**

EMG inactivity bout distribution is positively skewed. The density consists of a high number of short EMG inactivity bouts, and a lower number of long bouts. Typical metrics like mean or median do not characterize data well. Therefore, we followed calculation steps by Chastin and Granat[1]. First, each EMG inactivity bout and their duration was identified from data based on signal amplitude above the given EMG inactivity threshold. The bouts were ordered by ascending duration. Next, the cumulative sum of EMG inactivity duration was calculated for each EMG inactivity bout duration,

and these were scaled to be proportional to total EMG inactivity duration. The usual EMG inactivity bout duration was calculated using non-linear regression technique (Levenberg-Marquart), which can be used in automated processing for larger datasets. A sigmoid function $\frac{t^n}{(t^n + dW_{50\%}{}^n)}$ was fitted, where *t* is the EMG inactivity bout duration, *n* a free parameter, and $dW_{50\%}$ the usual EMG inactivity bout duration, above or below which 50% of EMG inactivity duration is accumulated. The non-linear regression techniques require initial starting parameters. The estimated model was plotted against data to visually confirm model fit for each individual, threshold and measurement. In case the preliminary starting parameters resulted in an inappropriate model or singular gradient matrix, different starting parameters were used case by case. This was often the case given very different accumulation pattern between the different thresholds used. For 1 μV threshold, for example, initial parameters X = 8 and n = 2 provided appropriate models, but this will be dependent on the dataset analyzed.

Results for sample size calculations

Tables S1 and S2 report results that can be used to calculate sample sizes for EMG inactivity duration and usual EMG inactivity bout duration in parallel and cross-over designs. Within-individual standard deviation was calculated according to Synek 2008 as follows[2]:

$$SD = \sqrt{\frac{\sum(X_{Con2} - X_{Con1})^2}{2n}}$$

where x_1 and x_2 are baseline and follow-up measurements, and n is number of observations, i.e. the number of x_1 and x_2 differences. Since the expected population mean is known (within control group equals 0), the sum of squares is divided by the number of differences, not by n – 1. Furthermore, because the squared standard deviation is twice that of individual results, the sum is divided by 2.

The within-individual coefficient of variation was calculated as the within-individual standard deviation divided by the mean:

$$CV\ (\%) = 100\ x\ \frac{SD}{Mean}$$

Table S1. Results for EMG inactivity duration sample size calculations

Threshold category	Thres hold	Intervention SD baseline	Pooled Intervention SD M1 and M2	Control within-individual SD	Control within-individual CV%	Control pre-post correlation
$\%EMG_{MVC}$	1%	8.87	10.52	6.00	9.07	0.74
	2%	8.01	9.44	5.36	7.15	0.69
	3%	7.56	8.69	4.82	5.96	0.63
	4%	7.19	8.08	4.44	5.22	0.58
Absolute	1µV	7.95	12.16	10.28	19.45	0.47
	2µV	7.88	9.94	6.64	10.56	0.69
	3µV	7.63	9.40	5.89	8.72	0.71
	4µV	7.36	8.94	5.53	7.78	0.71
Baseline sd	1sd	18.47	17.51	9.56	62.44	0.35
	2sd	20.09	20.45	10.58	32.04	0.66
	3sd	18.61	20.34	9.26	20.68	0.63
	4sd	16.81	18.59	8.31	15.97	0.56
Standing	0.6x	18.77	21.27	7.08	14.71	0.91
	0.7x	18.26	20.96	7.47	14.53	0.91
	0.8x	17.68	20.94	5.64	10.38	0.93
	0.9x	17.33	20.32	5.38	9.57	0.94

M1 = measurement 1, M2 = measurement 2.

Table S2. Results for usual EMG inactivity bout sample size calculations

Threshold category	Thres hold	Intervention SD baseline	Pooled Intervention SD M1 and M2	Control within-individual SD	Control within-individual CV%	Control pre-post correlation
$\%EMG_{MVC}$	1%	40.95	34.74	28.88	37.77	0.67
	2%	46.54	39.68	37.7	40.49	0.63
	3%	50.41	44.3	43.56	41.45	0.64
	4%	56.1	52.13	49.3	41.4	0.66
Absolute	1µV	22.77	20.26	27.29	68.1	0.43
	2µV	31.32	27.5	26.61	38.6	0.63
	3µV	38.49	32.76	29.16	37.53	0.65
	4µV	41.19	35.1	31.16	36.97	0.66
Baseline sd	1sd	9.29	9.9	2.49	87.55	0.59
	2sd	16.61	16.59	10.17	71.46	0.55
	3sd	22.5	21.84	12.5	44.3	0.78
	4sd	28.36	25.34	15.71	41.53	0.71
Standing	0.6x	39.63	35.64	31.68	69.08	0.53
	0.7x	40.74	36.46	32.44	64.7	0.53
	0.8x	42.54	37.82	32.98	60.75	0.53
	0.9x	42.02	38.11	31.44	54.2	0.58

M1 = measurement 1, M2 = measurement 2.

References

1. Chastin, S. F. M. & Granat, M. H. Methods for objective measure, quantification and analysis of sedentary behaviour and inactivity. *Gait Posture* **31**, 82–86 (2010).
2. Synek, V. Evaluation of the standard deviation from duplicate results. *Accreditation and Quality Assurance* **13**, 335–337 (2008).
